# Postoperative pain and quality of life after the use of different endodontic sealers in asymptomatic molars: a randomized clinical trial

**DOI:** 10.1007/s00784-025-06735-1

**Published:** 2026-01-10

**Authors:** Patrícia Santos Oliveira, Meire Coelho Ferreira, Natália Gomes de Paula, Wallace Vieira Mendes, Thieny Gouveia dos Santos, Giulio Gavini, Leonardo Hunaldo dos Santos, Suellen Linares Lima, Renata Grazziotin-Soares, Ceci Nunes Carvalho

**Affiliations:** 1 Dentistry Postgraduate Program, University Ceuma, R. Josué Montello, 1, Renascença II, São Luís, Maranhão 65075-120 Brazil; 2 Department of Restorative Dentistry, School of Dentistry, University of São Paulo (FOUSP), Av. Lineu Prestes, 2227, São Paulo, 05508-000 Brazil; 3 Health and Technology Postgraduate Program, Federal University of Maranhão (UFMA), R. Urbano Santos Street, Imperatriz, 65900-410 Maranhão Brazil; 4 School of Dentistry, Federal University of Maranhão (UFMA), Av. dos Portugueses, 1966, São Luís, 65080-805 Brazil; 5 Division of Endodontics, Dept. of Oral Biological and Medical Sciences, Faculty of Dentistry University of British Columbia, UBC Vancouver, Canada

**Keywords:** Endodontic sealer, Postoperative pain, Endodontics, Randomized clinical trial, Obturation, Quality of life

## Abstract

**Objective:**

To compare postoperative pain intensity and incidence after root canal obturation using AH Plus or EndoSequence BC in asymptomatic molars.

**Materials and methods:**

This randomized clinical trial included 152 patients and was conducted in accordance with CONSORT guidelines. Teeth were randomly allocated according to the sealer used. Postoperative pain was assessed using validated pain scales at 6, 12, and 24 h, and at 2 and 3 days after obturation. Quality of life was evaluated using the OHIP-14 questionnaire. Bite sensitivity and analgesic intake were also recorded.

**Results:**

Postoperative pain intensity was very low in both groups at all time points. The AH Plus group showed slightly higher pain intensity within the first 24 h compared with the BC sealer group (*p* < 0.05). No differences were observed between groups regarding pain incidence, bite sensitivity, or impact on quality of life (*p* > 0.05). Analgesic intake was higher in the AH Plus group. A weak positive correlation was observed between pain intensity and functional limitation domains of the OHIP-14 (physical disability and social impairment).

**Conclusions:**

Overall, both sealers resulted in very low postoperative pain. Although AH Plus showed slightly higher pain intensity compared with BC, both sealers demonstrated a similar risk of pain and comparable impact on quality of life.

**Clinical relevance:**

Postoperative pain after root canal obturation was minimal with both AH Plus and EndoSequence BC sealers. Minor differences in pain intensity were observed during the early postoperative period, with a slightly faster reduction in pain reported for EndoSequence BC, and no differences in pain risk or impact on quality of life.

**Supplementary Information:**

The online version contains supplementary material available at 10.1007/s00784-025-06735-1.

## Introduction

 Postoperative pain is a frequent complication of root canal treatment, with a reported prevalence ranging from 3% to 58%. Pain typically peaks within the first 24 h after obturation and gradually declines over the following 3–7 days [1–5]. Multiple factors contribute to post–root canal treatment pain, including preoperative pain, apical extrusion of debris, missed canals, overfilling, hyperocclusion, and the chemical properties of endodontic sealers [6–9].

Obturation is a critical phase for endodontic success, as it requires a three-dimensional seal, which is typically achieved using gutta-percha in combination with an endodontic sealer. Epoxy resin–based sealers, such as AH Plus (Dentsply Sirona, Switzerland), have traditionally been considered the gold standard, particularly for warm obturation techniques, due to their favorable physicochemical properties and biocompatibility. However, these materials lack bioactivity and may exhibit cytotoxic effects before setting [2, 7, 10–16].

Bioceramic sealers, such as EndoSequence BC Sealer (Brasseler, USA), have been introduced as alternatives. These calcium silicate–based materials exhibit bioactivity, antibacterial effects, and lower cytotoxicity compared with epoxy resin sealers, and are capable of forming hydroxyapatite bonds with dentin [17–20].

The literature remains inconsistent regarding the influence of endodontic sealers on post-obturation pain, partly because many studies fail to isolate the obturation phase from other treatment-related variables. Therefore, the aim of this study was to compare the occurrence and intensity of postoperative pain following obturation with EndoSequence BC Sealer versus AH Plus in asymptomatic molars up to 3 days after obturation. The null hypothesis was that the type of sealer would not influence postoperative pain.

## Materials and methods

### Study design and ethical approval

This study was designed as a randomized, parallel, blinded clinical trial and was conducted in accordance with the CONSORT 2010 guidelines [21]. The study was approved by the Research Ethics Committee of CEUMA University (Approval No. 3.813.690/2020). The study protocol was registered in the Brazilian Registry of Clinical Trials (ReBEC: U1111-1251-9531). No protocol deviations or modifications occurred after trial registration, in accordance with CONSORT requirements. All participants provided written informed consent. Treatments were performed between February 2021 and July 2024 at two locations: the university dental clinic in São Luís, Maranhão, Brazil, and a public health clinic in Açailândia, Maranhão, Brazil. Patients were blinded to the sealer used. Due to the distinct physical properties of the materials, the operator could not be blinded. The statistician was blinded to group allocation..

### Eligibility

Patients aged 18 to 66 years with permanent molars indicated for endodontic treatment were eligible if they were asymptomatic at baseline, diagnosed with pulp necrosis, asymptomatic irreversible pulpitis, or chronic hyperplastic pulpitis, and had adequate coronal structure to allow rubber dam isolation. Although limiting the sample to molars may have reduced the external validity of the data, molars represent the most anatomically complex teeth to treat and obturate. Therefore, the findings may help clinicians understand similar or less complex clinical situations, supporting result transferability.

Exclusion criteria were pregnancy; systemic contraindications to endodontic treatment (e.g., recent myocardial infarction, uncontrolled hypertension, or diabetes); preoperative use of analgesics or anti-inflammatory drugs; teeth with resorption, periodontal disease, or radiographically visible periapical lesions; anterior or premolar teeth; retreatment cases; spontaneous preoperative pain; orthodontically treated teeth; anatomical alterations or root curvature greater than 25°, assessed using the Schneider method [22]; uncooperative patients; or intolerance to nonsteroidal anti-inflammatory drugs..

### Allocation

Diagnosis was based on the chief complaint, medical and dental history, periapical radiographs, pulp sensitivity testing (Endo-Frost; Coltene-Whaledent, Germany), periodontal probing, palpation, and percussion. After diagnosis, eligible patients were invited by the researcher to participate in the study. Upon acceptance, patients were randomized using a block randomization scheme (block size = 4; Sealed Envelope™) to receive root canal obturation with EndoSequence BC Sealer (experimental group) or AH Plus (control group). Allocation concealment was ensured through the use of sealed opaque envelopes. The randomization sequence and preparation of the sealed envelopes were performed by one researcher, while envelope opening and patient allocation were carried out by another individual (a dental assistant). The operators were informed of the assigned endodontic sealer only at the obturation appointment..

### Sample size

Sample size was calculated to detect differences in mean postoperative pain scores with 95% confidence and 80% power, assuming mean pain scores of 2.7 (control) and 1.7 (experimental), SD = 2.2 [23]. A minimum of 76 patients per group (152 total) was required (Sealed Envelope™ power calculator)..

### Interventions

Treatments were performed in two visits by two trained endodontic specialists. Local anesthesia was administered using 2% lidocaine with 1:100,000 epinephrine (Alphacaine 1:100, Nova DFL, Brazil). Rubber dam isolation was performed, and access was obtained. Root canal scouting was performed using size 10 K-files (Dentsply Maillefer, Switzerland) with 2.5% sodium hypochlorite. Working length was determined using an electronic apex locator (Propex Pixi, Dentsply Sirona), set 1 mm short of the apical foramen, and confirmed radiographically. Instrumentation was performed with Reciproc instruments (R25, R40, R50; VDW, Germany) driven by a VDW Silver motor.

Reciproc instruments were selected due to their effectiveness, reduced working time, and postoperative pain levels comparable to those of rotary systems. Each file was used only once, with in-and-out motions of up to 3 mm. Irrigation was performed using a conventional syringe technique without agitation to reduce the potential risk of apical extrusion under positive pressure. A total of 15 mL of 2.5% sodium hypochlorite was used per tooth, delivered 2 mm short of the working length with a 30G side-vented needle. Final irrigation consisted of 2 mL of 17% EDTA for 3 min, followed by 2 mL of 2.5% sodium hypochlorite [24]. Root canals were dried using aspiration cannulas (Ultradent, USA) and paper points. Access cavities were sealed with glass ionomer cement (Vitro Fill LC, Nova DFL). No intracanal medication was used [25].

At the second visit, scheduled 7 days later, and after confirmation that the patient was asymptomatic, the temporary restoration was removed. The root canals were irrigated with 1 mL of 17% EDTA for 3 min and 10 mL of 2.5% sodium hypochlorite, and then dried. Obturation was performed using a single-cone cold technique with Reciproc gutta-percha cones and either EndoSequence BC Sealer or AH Plus. The excess filling material was seared off at the cementoenamel junction. The pulp chamber was cleaned with 70% alcohol, and the teeth were restored with a 2-mm layer of Tetric N-Flow Bulk Fill (Ivoclar Vivadent) at the canal orifices, followed by Filtek Z250 composite resin (3 M ESPE, USA). Standardized periapical radiographs were obtained to assess sealer extrusion..

### Outcome measures

Patients received forms containing: (1) two pain assessment scales—the Numeric Rating Scale (NRS; 0–10) and the Visual Analog Scale (VAS; 10 cm). Pain scores were categorized as 0 (none), 1–3 (mild), 4–6 (moderate), and 7–10 (severe); (2) a questionnaire assessing oral health–related quality of life—the Oral Health Impact Profile (OHIP-14), validated in Portuguese and provided as a take-home instrument, which evaluates seven domains using a 5-point Likert scale (0 = never to 4 = very often); and (3) a form to record bite sensitivity, in which patients were instructed to bite on a 3 × 2 cm latex device previously provided by the researchers at the treated tooth [26]. Outcomes were recorded at 6, 12, 24, 48, and 72 h postoperatively. One of the researchers was responsible for contacting patients by text message and/or phone call at each scheduled assessment time point to ensure completion of pain recordings. In cases of severe pain, ibuprofen (400 mg every 6 h) was prescribed, and patients were instructed to record the dosage and frequency of any medication taken.

### Data analysis

Descriptive statistics summarized demographic and clinical data. Associations between sealers and categorical variables (pain, bite sensitivity, OHIP-14 domains) were analyzed with chi-square or equivalent tests. Relative risk (RR, 95% CI) was calculated using AH Plus as reference. Repeated measures of pain (VAS) were analyzed with Generalized Estimating Equations (GEE) [27, 28] with Bonferroni post hoc tests for pairwise comparisons. Normality and homogeneity of variance were assessed with Kolmogorov-Smirnov and Levene’s tests. Since assumptions were not met, Mann-Whitney tests were applied to age and OHIP-14 scores. Spearman’s correlation tested associations between pain scales and quality-of-life domains. Analyses were performed with SPSS (IBM, USA) at 5% significance. Graphs were created in Microsoft Excel 365.

## Results

**Fig. 1:**
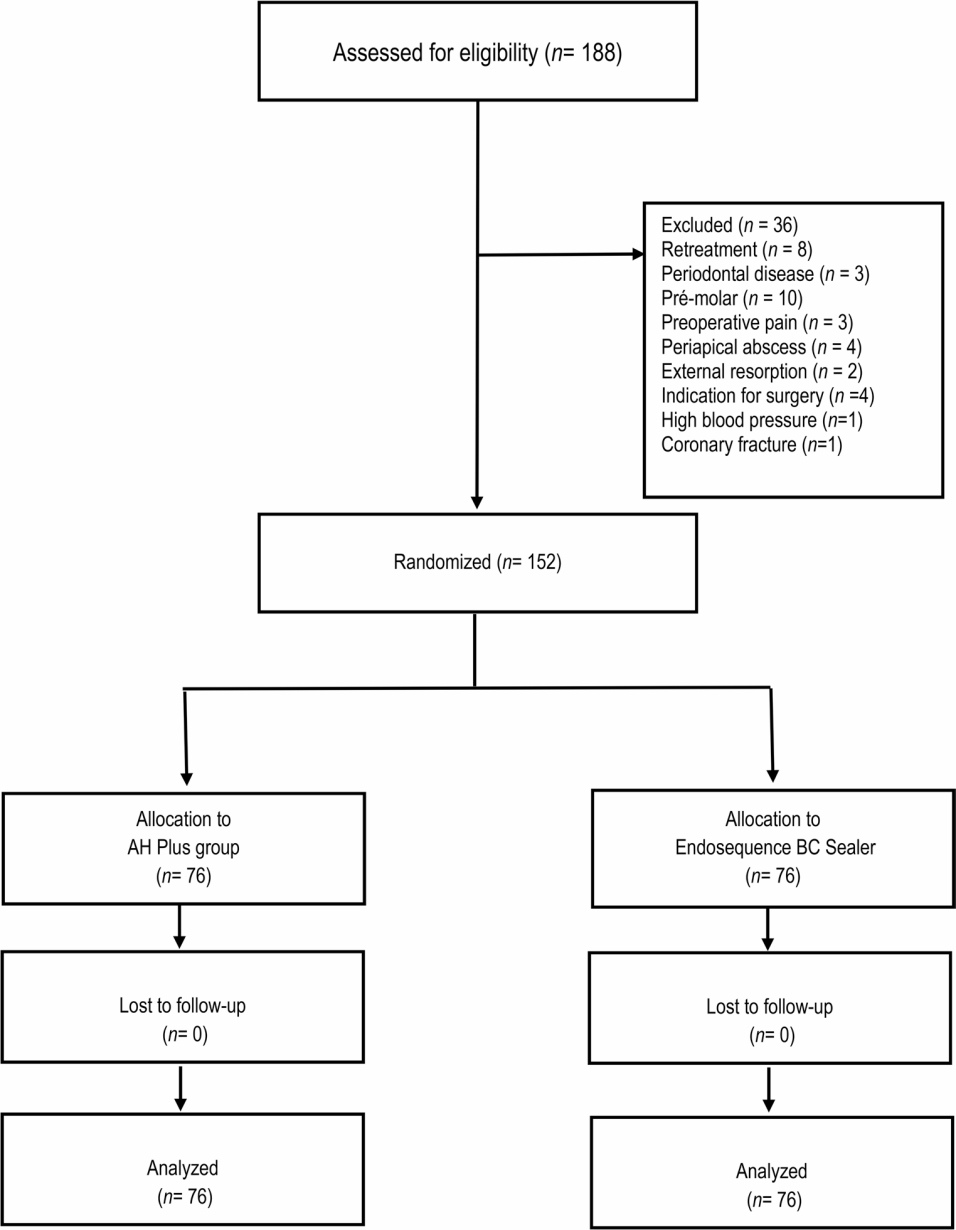
Flowchart of the clinical trial according to CONSORT

A total of 188 patients were assessed for eligibility; 36 were excluded for not meeting the inclusion criteria. The remaining 152 patients were randomized into two groups (AH Plus or BC Sealer), and all 152 patients were included in the final analysis (Fig. 1).

In the AH Plus group, 86% of the patients were diagnosed with asymptomatic irreversible pulpitis and 14% with pulp necrosis. In the EndoSequence BC Sealer group, 90.9% were diagnosed with asymptomatic irreversible pulpitis and 9.1% with pulp necrosis.

Among all included molars, 36.9% of maxillary teeth were allocated to the AH Plus group and 44.7% to the EndoSequence BC Sealer group (*p* > 0.5), while 63.1% of mandibular teeth were allocated to the AH Plus group and 55.3% to the EndoSequence BC Sealer group (*p* > 0.5). No statistically significant differences were found between groups regarding demographic, radiographic, or diagnostic characteristics (pulpitis vs. necrosis) (Table 1).

**Table 1:** Demographic, clinical, radiographic, and diagnostic characteristics in each group (*n* = 152)

Variable	AH Plus *n* (%)	BC Sealer *n* (%)	*p*-value
Sex			
Male	32 (42.1)	22 (29.0)	0.13*
Female	44 (57.9)	54 (71.0)	
Age	Mean (standard deviation)	31.2 (10.1)	32.5 (11.1)
Tooth			
Maxillary molar	28 (36.9)	34 (44.7)	0.41*
Mandibular molar	48 (63.1)	42 (55.3)	
Pulp vitality test			
Positive	60 (78.9)	68 (89.5)	0.12*
Negative	16 (25.1)	8 (10.5)	
Radiographic aspect of the periapical region			
Normal periodontal ligament space	72 (94.7)	72 (94.7)	1.00*
Widened periodontal ligament space	4 (5.3)	4 (5.3)	
Clinical diagnosis			
Asymptomatic irreversible pulpitis	65 (85.6)	69 (90.9)	0.23**
Pulp necrosis	11 (14.4)	7 (9.1)	
Number of canals			
2 canals	3 (3.9)	4 (5.2)	0.60**
3 canals	63 (82.9)	58 (76.4)	
4 canals	10 (13.2)	14 (18.4)	

* Chi-square test ** Fisher’s exact test *** Student’s t-test

**Fig. 2:**
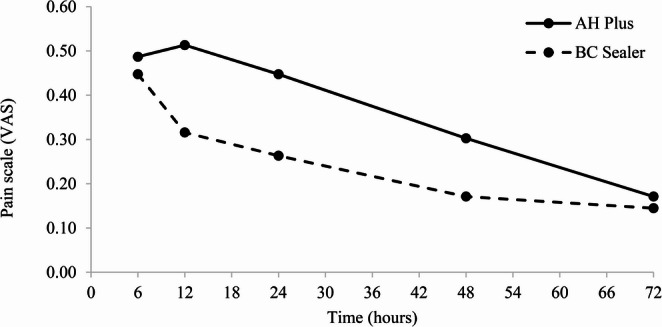
Average post obturation pain intensity (VAS) between groups

For both sealers, the NRS (0–10) and VAS showed a strong positive correlation (AH Plus: Spearman’s ρ = 0.909, *p* < 0.001; BC Sealer: ρ = 0.912, *p* < 0.001). Therefore, only postoperative pain analyses based on the VAS were presented, as it is the most widely used tool for pain assessment in clinical trials [7, 23]. Table 2 presents the mean scores and standard deviations (SD) of postoperative pain intensity (VAS) for each group at the different time points. The highest mean pain intensity (pain peak) occurred within the first 24 h in the AH Plus group, whereas in the BC Sealer group the peak occurred within the first 6 h after obturation. A reduction in pain intensity began on the second day after obturation in the AH Plus group, whereas in the BC Sealer group the reduction started at 12 h (*p* = 0.04). Significant differences in postoperative pain intensity were observed between the groups at 12 h, 24 h, and on day 2. Figure 2 presents the mean postoperative pain intensity for AH Plus and BC Sealer at the different time points. Overall, postoperative pain in both groups was of low intensity, with mean pain scores close to zero at all evaluated time points.

**Table 2:** Mean scores and standard deviations of pain intensity, from the VAS scale, for the groups, at various time intervals after obturation

Groups	6 h	12 h	24 h	Day 2	Day 3	*p*-value*
**AH Plus** Mean (SD)	0.48 (0.87)a	0.51 (0.94)a	0.44 (0.77)a	0.30 (0.63)b	0.17 (0.45)c	*< 0.001*
**BC Sealer** Mean (SD)	0.44 (0.83)a	0.31 (0.70)b	0.25 (0.64)bc	0.17 (0.50)c	0.15 (0.46)c	*< 0.001*
p-value*	*0.48*	*0.04*	*0.02*	*0.001*	*0.75*	

SD – Standard deviation. *Wald chi-square (Generalized Estimating Equation Models - GEE). Means with different letters in the same row are statistically different by the Bonferroni test at 5% significance.

**Fig. 3:**
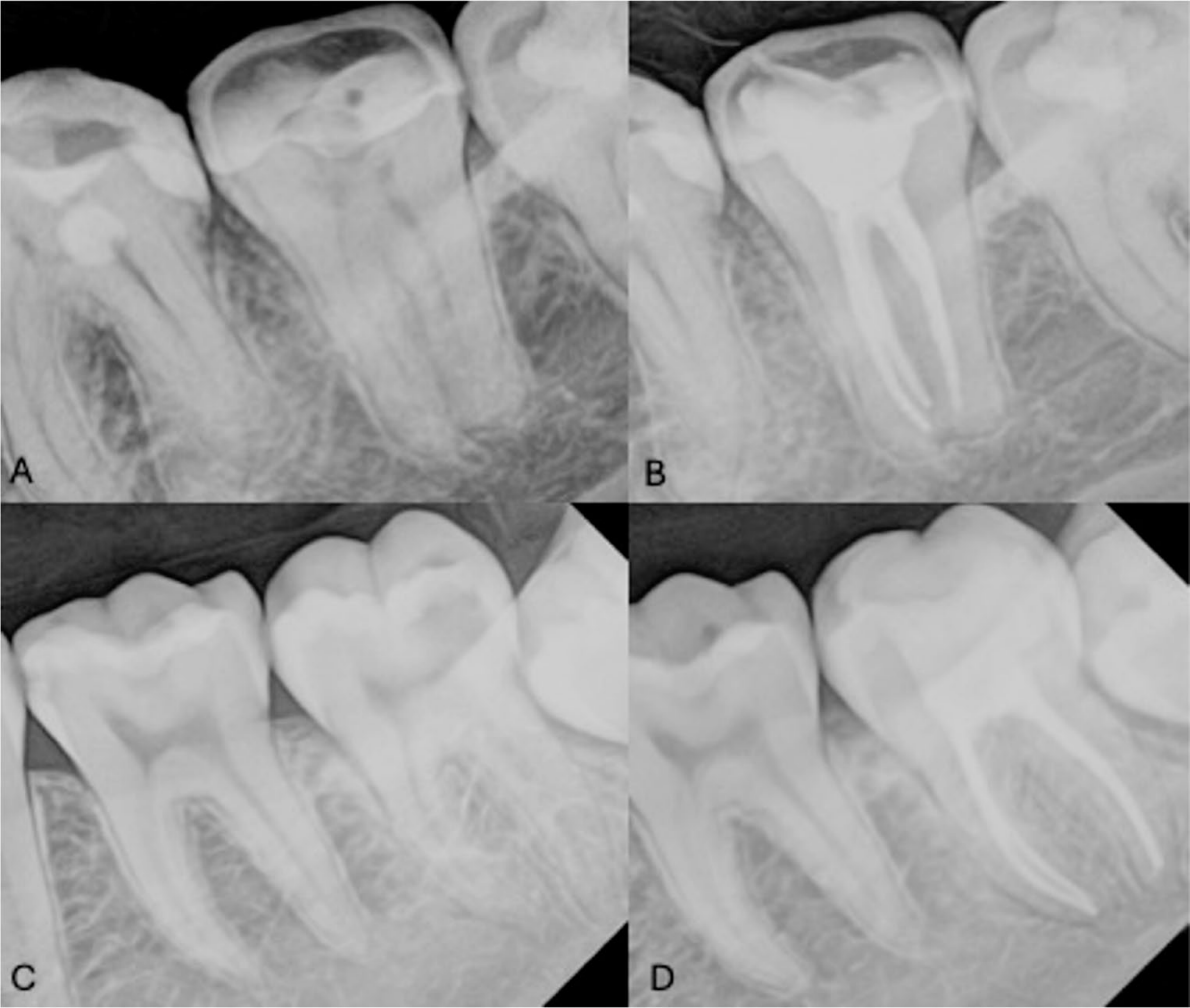
Representative clinical cases from the two obturation groups. (**A**) Asymptomatic molar before endodontic treatment. (**B**) Same tooth after root canal obturation using the single-cone cold technique with EndoSequence BC Sealer. (**C**) Asymptomatic molar before endodontic treatment. (**D**) Same tooth after root canal obturation using the single-cone cold technique with AH Plus sealer

Table 3 shows that most participants did not report pain after obturation with either sealer, and no statistically significant differences were found between groups regarding the absolute frequency or risk of postoperative pain. Regarding bite sensitivity, assessed using a latex device, no significant differences were observed between groups or across time intervals (Table 4). With respect to analgesic intake, 24 patients in the AH Plus group and 11 in the BC Sealer group reported taking pain medication, mostly within the first 24 h, resulting in a statistically significant difference in analgesic consumption between groups (*p* = 0.02). The mean number of tablets taken for pain relief was 2.46 ± 1.9 in the AH Plus group and 2.5 ± 3.3 in the BC Sealer group. Sealer extrusion, as visualized on radiographs, occurred in three cases in the AH Plus group and one case in the BC Sealer group. In all cases, visible extrusion did not exceed 1 mm (Fig. 3).

**Table 3:** Frequency and risk of pain (VAS) at different times after obturation: comparison between AH plus and BC sealer

Time	Obturation	Post op Pain				RR (95% CI)*	p-value**
		No		Yes			
		N	%	N	%		
6 h	AH Plus	53	69.7	23	30.3	1.00	*0.72*
	BC Sealer	55	72.4	21	27.6	0.96 (0.79–1.18)	
12 h	AH Plus	55	72.4	21	27.6	1.00	*0.45*
	BC Sealer	60	78.9	16	21.1	0.92 (0.77–1.10)	
24 h	AH Plus	54	71.1	22	28.9	1.00	*0.12*
	BC Sealer	63	82.9	13	17.1	0.86 (0.72–1.02)	
Day 2	AH Plus	59	77.6	17	22.4	1.00	*0.13*
	BC Sealer	67	88.2	9	11.8	0.88 (0.76–1.02)	
Day 3	AH Plus	67	88.2	9	11.8	1.00	*1.00*
	BC Sealer	68	89.5	8	10.5	0.99 (0.88–1.10)	

*Calculated based on the presence of pain. **Chi-square test with Yates correction.

**Table 4:** Bite sensitivity at different times after filling: comparison between AH plus and BC sealer

Time	Obturation	Bite sensitivity				RR (95% CI)*	p-value**
		No		Yes			
		n	%	n	%		
6 h	AH Plus	62	81.6	14	18.4	1.00	*0.69*
	BC Sealer	59	77.6	17	22.4	1.05 (0.89–1.24)	
12 h	AH Plus	60	78.9	16	21.1	1.00	*0.68*
	BC Sealer	63	82.9	13	17.1	0.95 (0.82–1.11)	
24 h	AH Plus	63	82.9	13	17.1	1.00	*0.82*
	BC Sealer	65	85.5	11	14.5	0.97 (0.84–1.11)	
Day 2	AH Plus	62	81.6	14	18.4	1.00	*0.37*
	BC Sealer	67	88.2	9	11.8	0.93 (0.81–1.06)	
Day 3	AH Plus	68	89.5	8	10.5	1.00	*1.00*
	BC Sealer	68	89.5	8	10.5	1.00 (0.40–2.53)	

*Calculated based on bite sensitivity. **Chi-square test with Yates correction.

No significant differences were found in the quality-of-life parameters after obturation between groups (Table 5), suggesting that the endodontic sealers had similar effects. Table 6 shows the mean scores and standard deviations of quality-of-life domains (OHIP-14) in both groups. For the AH Plus group, moderate to weak positive correlations were observed between mean pain intensity (VAS) and the OHIP-14 domains of functional limitation, physical pain, physical disability, psychological disability, and social disability (Table 7). For the BC Sealer group, moderate positive correlations were observed between mean pain intensity (VAS) and the OHIP-14 domains of functional limitation, physical pain, psychological discomfort, physical disability, psychological disability, and social disability (Table 8).

**Table 5:** Frequency of impact on quality of life after root Canal filling with AH plus and BC sealer (*n* = 152) table continue

	**AH Plus**		**BC Sealer**		*p-value*
	**n**	**(%)**	**n**	**(%)**	
Functional Limitation					
Had trouble speaking					
Never	71	93.4	69	90.8	*0.54*
Rarely	1	1.3	3	3.9	
Sometimes	4	5.3	3	3.9	
Often	0	0	1	1.3	
The taste of food has become worse					
Never	70	92.1	69	90.8	*0.17*
Rarely	6	7.9	3	3.9	
Sometimes	0	0	3	3.9	
Often	0	0	1	1.3	
Always	-	-	-	*-*	
Physical Pain					
Have you experienced pain in your mouth?					
Never	53	69.7	58	76.3	*0.40*
Rarely	11	14.5	8	10.5	
Sometimes	8	10.5	8	10.5	
Often	3	3.9	0	0	
Always	1	1.3	2	2.6	
Have you felt discomfort when eating?					
Never	55	72.4	59	77.6	*0.79*
Rarely	8	10.5	4	5.3	
Sometimes	10	13.2	9	11.8	
Often	2	2.6	3	3.9	
Always	1	1.3	1	1.3	
Psychological Discomfort					
Was worried about the tooth					
Never	58	76.3	53	69.7	*0.91*
Rarely	5	6.6	7	9.2	
Sometimes	9	11.8	10	13.2	
Often	2	2.6	3	3.9	
Always	2	2.6	3	3.9	
Felt stressed about the tooth					
Never	65	85.5	63	82.9	*0.52*
Rarely	5	6.6	4	5.3	
Sometimes	5	6.6	9	11.8	
Often	1	1.3	0	0	
Always	-	-	-	*-*	
Physical Disability					
Eating was impaired					
Never	67	88.2	64	84.2	*0.37*
Rarely	4	5.3	3	3.9	
Sometimes	4	5.3	9	11.8	
Often	1	1.3	0	0	
Always	-	-	-	*-*	
Had to stop eating					
Never	69	90.8	67	88.2	*0.49*
Rarely	5	6.6	4	5.3	
Sometimes	2	2.6	5	6.6	
Often	-	-	-	*-*	
Always	-	-	-	*-*	
Psychological Disability					
Had difficulty relaxing					
Never	69	90.8	65	85.5	*0.76*
Rarely	2	2.6	4	5.3	
Sometimes	4	5.3	6	7.9	
Often	1	1.3	1	1.3	
Always	-	-	-	*-*	
Felt embarrassed about tooth					
Never	73	96.1	68	89.5	*0.17*
Rarely	1	1.3	2	2.6	
Sometimes	1	1.3	6	7.9	
Often	-	-	-	*-*	
Always	1	1.3	0	0	
Social Impairment					
Became irritable with other people					
Never	74	97.4	70	92.1	*0.41*
Rarely	1	1.3	1	1.3	
Sometimes	1	1.3	4	5.3	
Often	0	0	1	1.3	
Always	-	-	-	*-*	
Had difficulty performing daily activities					
Never	68	89.5	71	93.4	*0.32*
Rarely	2	2.6	3	3.9	
Sometimes	6	7.9	2	2.6	
Often	-	-	-	*-*	
Always	-	-	-	*-*	
Social Disadvantage					
Felt that life had become worse					
Never	73	96.1	72	94.7	*0.14*
Rarely	3	3.9	1	1.3	
Sometimes	0	0	3	3.9	
Often	-	-	-	*-*	
Always	-	-	-	*-*	
Was completely unable to carry out daily activities					
Never	75	98.7	74	97.4	*1.00*
Rarely	-	-	-	*-*	
Sometimes	1	1.3	2	2.6	
Often	-	-	-	*-*	
Always	-	-	-	*-*	

*Chi-square test*

**Table 6:** Quality of life domains between groups (*n* = 152)

	AH Plus		BC Sealer		*p*-value
	Mean	SD	Mean	SD	
Functional limitation	0.20	0.59	0.33	0.93	*0.58*
Physical pain	1.03	1.69	0.88	1.76	*0.35*
Psychological discomfort	0.72	1.47	0.92	1.66	*0.45*
Physical disability	0.32	0.90	0.46	1.12	*0.47*
Psychological disability	0.26	0.85	0.43	1.11	*0.33*
Social disability	0.22	0.69	0.25	0.73	*0.81*
Social disadvantage	0.07	0.38	0.14	0.60	*0.46*

*Mann Whitney test*

**Table 7:** Correlation between quality-of-life domains and average pain for AH plus (*n* = 152)

			Mean Level of Pain (VAS)
Functional limitation	(ρ)	0.26	
	*p-value*	*0.02*	
Physical pain	(ρ)	0.44	
	*p-value*	*< 0.001*	
Psychological discomfort	(ρ)	0.19	
	*p-value*	*0.10*	
Physical disability	(ρ)	0.39	
	*p-value*	*< 0.001*	
Psychological disability	(ρ)	0.25	
	*p-value*	*0.03*	
Social disability	(ρ)	0.30	
	*p-value*	*0.01*	
Social disability	(ρ)	0.22	
	*p-value*	*0.06*	

*Spearman correlation (ρ).*

**Table 8:** Correlation between quality-of-life domains and average pain for BC sealer (*n* = 152)

		Mean Level of Pain (VAS)
Functional limitation	(ρ)	0.24
	*p-value*	*0.04*
Physical pain	(ρ)	0.55
	*p-value*	*< 0.001*
Psychological discomfort	(ρ)	0.36
	*p-value*	*< 0.001*
Physical disability	(ρ)	0.53
	*p-value*	*< 0.001*
Psychological disability	(ρ)	0.53
	*p-value*	*< 0.001*
Social disability	(ρ)	0.52
	*p-value*	*< 0.001*
Social disability	(ρ)	0.24
	*p-value*	*0.04*

*Spearman’s correlation (ρ).*

## Discussion

Interappointment postoperative dental pain may reflect transient inflammation, a foreign body reaction, or an extraradicular infection, leading to pain or edema. In contrast, post-obturation pain is of greater concern because the source of inflammation can no longer be removed. The present study investigated post-obturation pain potentially associated with the composition of endodontic sealers and the root canal obturation techniques used [23].

Pain is a subjective outcome that varies among individuals and is influenced by personal expectations, cultural and socioeconomic factors [2], as well as by different dental techniques [29]. With this in mind, the present clinical trial was designed to minimize the influence of confounding variables by focusing specifically on postoperative pain related to root canal obturation. Canal preparation and irrigation protocols were standardized to reduce operator- and technique-dependent variability. In addition, post-obturation pain was evaluated independently from pain related to canal preparation.

All participants’ molars were treated in two sessions with a 7-day interval. During the first session, access cavity preparation, cleaning, working length determination, and canal preparation were performed. The root canals were dried, no intracanal medication was placed, coronal sealing was performed, and occlusal adjustment was carried out. At the second session, patients were free from post-preparation pain and obturation was subsequently performed. Therefore, potential post-instrumentation pain related to inflammatory responses caused by extruded irritants, such as debris or irrigants [26], was not a confounding factor in this study.

To further reduce confounding factors related to individual characteristics [1, 27], only asymptomatic molars without periapical pathology were included. Accordingly, the two study groups showed similar demographic, radiographic, and diagnostic characteristics (*p* > 0.05).

Strengths of this clinical study include an adequate sample size, randomization, patient blinding, standardized endodontic procedures, and treatment performed by only two operators. The potential influence of conducting treatments in two different clinical settings was mitigated by ensuring that both centers followed identical protocols, that operators were calibrated, and that all procedures were performed under similar clinical conditions, minimizing the risk of center-related bias. Consequently, the results reflect the use of both sealers in routine clinical practice.

High internal validity was achieved through effective randomization, which resulted in homogeneous groups and minimized the influence of individual characteristics on outcomes. Additional strengths include patient blinding to the endodontic sealer and the self-administration of the NRS and VAS scales. Pain responses were standardized and recorded using forms provided at the end of each session, reducing potential researcher influence and minimizing measurement bias. Root canal obturation was performed in accordance with the manufacturers’ instructions.

Previous studies on post-obturation pain have reported variable results. A split-mouth study comparing AH Plus and Total Fill in single-rooted teeth with asymptomatic apical periodontitis reported postoperative pain in 35% of patients, with no significant differences in VAS scores between groups [1]. Another study involving anterior teeth and premolars with pulp necrosis treated in two sessions reported postoperative pain in only 11.6% of patients [30]. These findings support the use of a two-session protocol to isolate post-obturation pain from the effects of canal preparation, as adopted in the present study.

A systematic review and meta-analysis found no significant differences in post-obturation pain between epoxy resin–based and calcium silicate–based sealers, although calcium silicate sealers tended to be associated with slightly lower pain levels after 48 h [31]. In the present study, AH Plus was associated with higher pain intensity during the first 24 h, with a gradual reduction by day 3, whereas pain intensity with EndoSequence BC peaked at 6 h and decreased significantly after 12 h. Despite these statistically significant differences, pain scores were consistently very low, indicating limited clinical relevance.

Because endodontic sealers may come into direct or indirect contact with periradicular tissues, biocompatibility is a critical factor [32]. Postoperative pain has generally been reported to be similar between premixed bioceramic sealers (e.g., iRoot, TotalFill, EndoSequence BC) and epoxy resin–based sealers such as AH Plus [1, 23, 33]. The cytotoxicity of endodontic sealers may vary over time and depends on material concentration, which may explain differences in cell viability observed at 24 and 48 h. In a laboratory study [34], BioRoot showed reduced cell viability at 24 h, whereas no differences were observed after 48 h compared with calcium silicate–based, epoxy resin–based, and zinc oxide–eugenol sealers [35]. In addition, bioactive sealers have demonstrated the ability to minimize acute inflammatory responses and promote faster periapical healing [36].

According to Zamparini et al. (2023) [37], the morphology and extent of sealer extrusion are influenced by root diameter and, most likely, by the presence of a periapical lesion. In the present study, all treated teeth had fully formed apices and no radiographic evidence of periapical lesions, which may have contributed to the limited extent of sealer extrusion observed.

The use of two pain assessment scales (NRS and VAS) allowed a more accurate evaluation of self-reported pain [38]. Nevertheless, individual variability in pain thresholds may still have influenced patients’ responses. Ibuprofen (400 mg every 6 h) was prescribed only in cases of severe pain, in accordance with recommendations for the management of post-endodontic pain [1, 4, 5, 7, 11]. Although a statistically significant difference in analgesic intake was observed between groups, overall analgesic consumption was low and limited to the early postoperative period. Previous evidence suggests that analgesic intake may partially mask reported pain scores, representing a limitation of pain assessment [23].

Periradicular inflammation was assessed using a latex bite device simulating a vertical percussion test previously described in clinical research [26]. This approach enabled home-based monitoring across time points. No significant differences were observed between sealer groups, with percussion-related pain being more frequent during the first two days and decreasing by the third day, consistent with NRS and VAS findings.

Post-obturation pain may affect patients’ quality of life [13, 39]. Although no differences were observed between groups regarding overall quality-of-life outcomes, increasing pain intensity was associated with negative effects on specific domains. For the AH Plus group, affected domains included functional limitation, physical pain, physical disability, psychological disability, and social disability. For the EndoSequence BC group, affected domains included functional limitation, physical pain, psychological discomfort, physical disability, psychological disability, and social disability.

Some limitations of this study should be acknowledged. Pain is a subjective outcome and may be influenced by individual pain thresholds and the use of analgesics, which may partially mask reported pain scores. In addition, only asymptomatic molars without periapical lesions were included, which may limit the external validity of the findings. Irrigant activation techniques and different instrumentation systems were not evaluated, as these variables were intentionally standardized to isolate the effect of the endodontic sealers on postoperative pain. Furthermore, although treatments were performed in two clinical settings, strict protocol standardization and operator calibration were adopted to minimize center-related bias. Despite these limitations, the randomized design and methodological standardization strengthen the internal validity of the study.

## Conclusion

Overall, both sealers resulted in very low postoperative pain. Although AH Plus was associated with slightly higher pain intensity during the first 24 h and a longer time to return to baseline, EndoSequence BC showed an earlier pain peak with faster resolution. Despite these minor differences in pain intensity, both sealers demonstrated a similar risk of postoperative pain and a comparable impact on quality of life.

## Supplementary Information

Below is the link to the electronic supplementary material.

Supplementary Material 1 (DOC 218 KB)

## Data Availability

All the research data used in this manuscript will be available whenever requested.
